# Evaluation of sodium valproate low dose efficacy in radicular pain management and it’s relation with pharmacokinetics parameters

**DOI:** 10.37796/2211-8039.1039

**Published:** 2020-09-01

**Authors:** Mona Ghasemian, Mohammad Bagher Owlia, Mohammad Hossein Mosaddegh, Masoud Nakhaie nejad, Seyed Mojtaba Sohrevardi

**Affiliations:** a Department of Clinical Pharmacy, Shahid Sadoughi University of Medical Sciences, Yazd, Iran; b Department of Internal Medicine, Shahid Sadoughi University of Medical Sciences, Yazd, Iran; c Department of Toxicology, Shahid Sadoughi University of Medical Sciences, Yazd, Iran; d Department of Pharmaceutical Sciences, Shahid Sadoughi University of Medical Sciences, Yazd, Iran

**Keywords:** Low dose, Pharmacokinetic, Radicular pain, Sodium Valproate, Anti-convulsant

## Abstract

**Background:**

Radiculopathy due to lumbar or cervical disc disease is the most common chronic neuropathic pain in adults. The aim of present study was evaluation of low dose of sodium valproate (VPA) on radicular pain and determining VPA pharmacokinetics.

**Materials and Methods:**

In this double blind randomized placebo control clinical study, 80 patients with established lumbar or cervical radicular pain, have been randomly allocated into two study groups: 40 have received sodium valproate 200 mg/day and Celecoxib 100 mg/day and acetaminophen 500 mg PRN as rescue medication, and second group has received placebo, Celecoxib and acetaminophen. Quantitative assessment of pain was done by visual analogue scale (VAS) prior to perform the intervention and after ten days (treatment duration). Blood sample has been taken for determining mean through concentration after five half-lives. Evaluation of plasma concentration of VPA and that of efficacy on pain score relationship by comparing VAS before and after the therapy was done.

**Results:**

Group A and B have demonstrated significant alleviation in mean VAS score; −21.97 ± 25.41, −14.39 ± 23.03 respectively (P < 0.001). The mean plasma concentration of VPA in group A was: 26.9 ± 13.5 mg/L. Moreover, no significant correlation was seen between pain score with age, gender, and weight (p > 0.05).

**Conclusion:**

Low dose of sodium valproate especially together with NSAIDs demonstrated good efficacy in lumbar and cervical radicular pain management.

## 1. Introduction

Radicular pain is the main manifestation of radiculopathy that lancinating and traveling on a narrow band which it maybe repetitive episode or paroxysmal. The main cause of this form of pain is ectopic activation of ischemic damaged nerve roots or inflamed dorsal root ganglion [1, 2]. The chief complain of cervical and lumbar radicular pain is radiated knife like shooting pain in shoulder to arm and leg to foot respectively [3, 4]. Cervical and lumbar radicular pain is often correlated with neurologic symptoms comprise of numbness, lacks of sensitivity reflexes and motor weakness called radiculopathy [4]. Although lumbar radicular pain owing to damaged disc is at least 20 times more common than other neuropathic pains with 4.5% prevalence in adults older than 30 years old, there are no analgesic dose-control trials in this disease [5]. It is essential to consider that other neuropathic syndrome such as diabetic and post-herpetic neuralgia have different site and pathology and generalizing those research outcomes to radiculopathy seems to be inappropriate [5]. Untreated radiculopathy can lead to disablement and impose a huge economic burden on individuals and society. Through various treatments have been applied, anti-convulsant drugs have demonstrated satisfactory results to pain relieving in radicular pain patients. Sodium valproate (VPA) is an anti-convulsant medicine which has proved to be efficient in migraine prophylaxis and trigeminal neuralgia, diabetic neuropathy [6, 7] and chronic central pain after spinal cord injury [8]. The main mechanism of action has been proposed for valproate is prevention in degradation and neuronal uptake of Y-amino butyric acid (GABA) as an inhibitory neurotransmitter in central nervous system[9]. In recent studies the maximum possible dose of sodium valproate has been dispensed for neuropathic pain relieving [6–8]. Though the available evidence for that of minimum effective dose is not enough and sufficient. Themain goals of this present study are evaluation of effectiveness of low dose of VPA in Iranian radicular pain patients and correlation of trough concentration of VPA with visual analog scale (VAS).

## 2. Material and methods

**Fig. 1:**
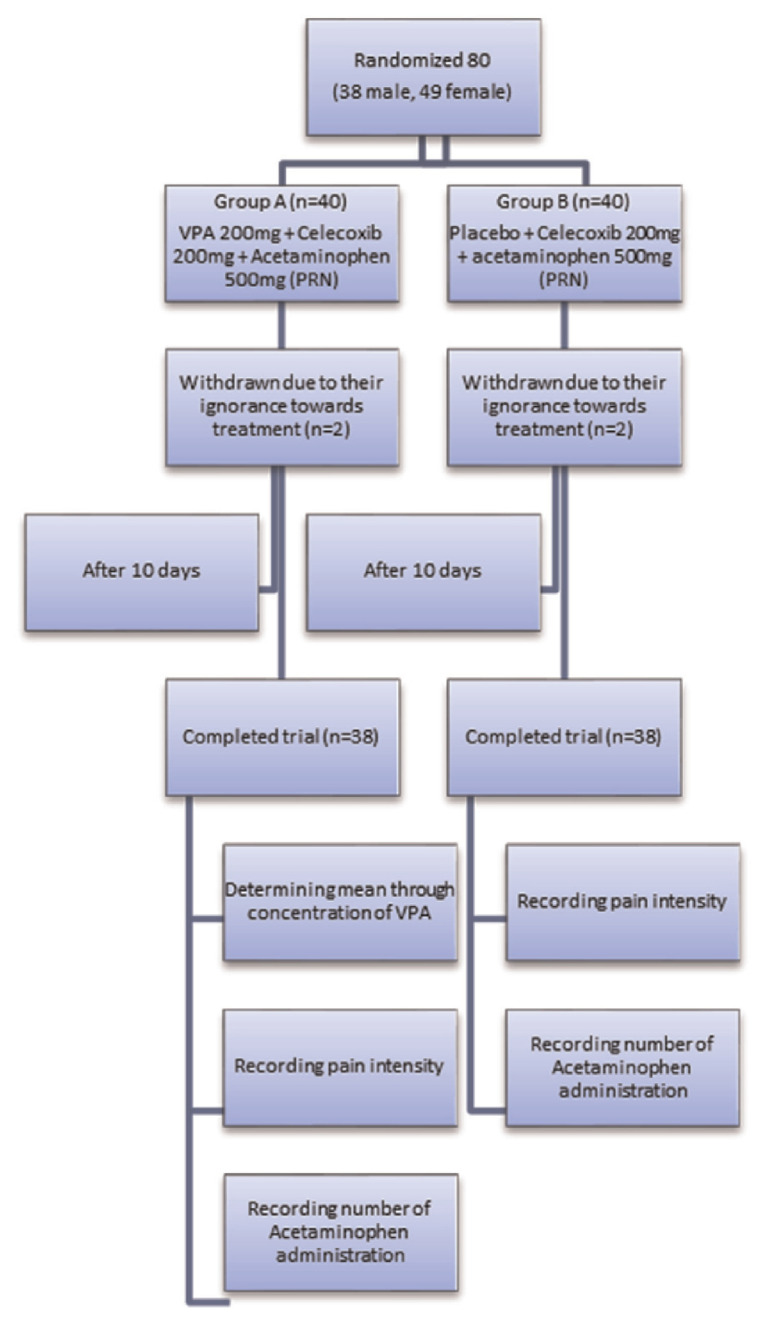
Consort Diagram.

The present double-blind randomized placebo controlled study was conducted in 80 patients (35 male, 45 female) who have suffered from cervical and or lumbar radicular pain (Fig. 1). 20–70 years old subjects without any certain medical complication such as gestation, heart failure, liver and or kidney disease had been enrolled for the trial, likewise patients who have taken non-steroidal anti-inflammatory drugs, anti-convulsants for radicular pain relief or other medical reasons and any drugs which had had direct or indirect impress on patients subjective pain insight (SSRI or TCA and other anti-depressants) were excluded or if it was possible the patients were asked to stop drugs at least one week before study.

Diagnosis was based on history of the symptoms and physical examination, straight leg raise test have been used to recognize a lumbar radiculopathy by rheumatologist.

The selected Patients had been allowed time to ask questions before written informed consent was taken.

All patients were randomly divided into 2 groups, randomization was done by the use of random numbers table, treatment plan of group A comprised of sodium valproate 200 mg/day, Celecoxib 200 mg/day and acetaminophen 500 mg PRN (acetaminophen, up to 2000 mg/day was allowed as the rescue medication), group B or control group’s treatment plan contained placebo once a day, Celecoxib 200 mg/day and acetaminophen 500 mg PRN, duration of treatment was ten days, meanwhile all patients were emphasized that Valproate must be administered on 12 AM before meal and Celecoxib must be administered at night after dinner, except for treatment with sodium valproate, target therapy groups and control group were similar in all respects. The placebo tablets were identical in shape and color with that of valproate sodium tablets. All patients, who had been enrolled for group A, took valproate tablets in the same batch number from the same manufacturer company. All patients were monitored for adverse events (gastrointestinal disorders, allergic reaction, and somnolence and …) during the course of treatment. Each patient was rated his/her pain intensity on the visual analog scale (VAS) from 0–100 (0 = no pain, 100 = worst pain imaginable) before beginning and after the end of intervention (VAS assessment occurred at baseline and day ten). The number of consuming acetaminophens as a rescue medication was recorded in a personal diary during ten days by each subjects and was documented in individuals relevant information file after the end of therapy by the member of research team. After collecting data, all of them were evaluated by blinded statistician and then the outcomes were decoded and divided the same into target therapy group (A), group (B) and then were compared to each other.

Inclusion criteria: 1. Diagnosis of lumbar or cervical radicular by the rheumatologist. 2. Age between 20 and 70 years. 3. No possibility of health risk or confounding by other diseases, e.g., pregnancy, hepatic or kidney disorders, congestive heart failure. 4. Normal liver function 5. Patients hadn’t used valproate sodium or if they had been using, they must remember pain intensity before using valproate sodium and can compare with current pain intensity. 6. Weight between 50 and 90 kg. Exclusion criteria: 1. Intolerance to sodium valproate side effects. 2. Allergic history to VPA or NSAIDs. 3. Patients would be forced to administered medicines that change protein binding or clearance of valproate sodium. 3. Patients would be given diseases that affect the results of study.

Gas chromatography method.

### 2.1. Sample preparation

All blood samples were stored in −20°C till analyzed. 0.5 ml of each serum was separated and four drops perchloric acid 60% was added in order to protein precipitation and achievement to desire PH (1), this was followed by five min shaking to complete deproteination, after centrifugation at 3000 rpm for ten min the supernatant fluid was transferred to a ten ml volumetric flask, after 20 min break time (in order to complete acidification process) deionized water was added to ten ml gradually, this samples were stored in 2–8°c following 3 min mixing until analyzed.

### 2.2. Spiking solutions and calibration curve

The serial dilution of spiking solutions were made up by adding the different amounts of the stock solution (10 mg/ml) to 0.5 ml drug free serum, deproteinating and the rest of process have been done according what was mentioned in sample preparation method, diluting with deionized water up to ten ml in order to obtain different concentrations from 2–100 μg/ml (2, 5, 10, 20, 40, 50, 100) was performed. All spiking samples were analyzed by gas chromatography/flame ionization detector (GC/ FID) in the same constant thermal ramping with the unknown samples, calibration curve and linear regression were performed then the Y-intercept and the slope of plotted curve were calculated and replaced in linear equation.

### 2.3. Extraction method

Analyte have been extracted by solid phase micro extraction (SPME) method. For creating a partitioning between the sample matrix and the fibre coating the needle of SPME set was passed through the septum which sealed sample vial and the polyacrylate fibre was entered to headspace above the sample in this meantime the glass vial of sample was heated and stirred by the hotplate Labtron model L-50; magnet stirrer speed was set on point one and temperature on 60°C. sample extraction and desorption time were 20 and five min respectively.

## 3. Results

**Fig. 2:**
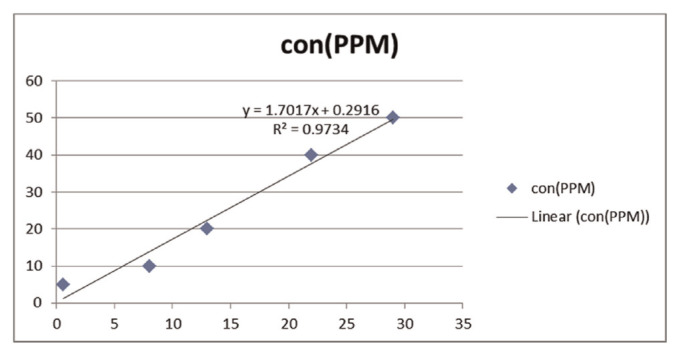
Calibration curve.

All groups were similar in demographic features like mean age, mean weight, and primary and secondary pain scores as summarized in Table 1. Distribution of males and females was identical among the groups. The mean age of patients in group B was a bit greater than the other group. The patients with radicular cervical and lumbar pain showed a similar scattering pattern. The difference in the patients’ primary pain scores was not significant among the two groups. Two patients in each of Group A and Group B did not present to laboratory for follow-up because they did not consume the drugs due to their ignorance towards treatment. The area under the curve (AUC) was converted to concentration through calibration curve line equation obtained from Fig. 2 where Y represents plasma concentration and X is the area under curve. According to what was explained under “Methodology”, the parameters of trough concentration (Conc), elimination constant (Ke), true clearance (true Cl), volume distribution (Vd), and true half-life were calculated for each patient as given in Table 2. The population elimination constant was estimated by considering 8 mg/kg/h clearance and population distribution volume 0.14 L/Kg using formula (1) which is constant for all patients.

Formula 1:

(1) $$\begin{array}{l} Cl\left(\frac{lit}{hr}\right)=Vd(lit)ke\left(\frac{1}{hr}\right)\\ \text{Ke population }(1/\text{h})=0.057. \end{array}$$

Similarly, the population half-life for valproate sodium was calculated using formula (2), a parameter which is again constant for all patients.

Formula 2:

(2) $$\begin{array}{c} t\;1/2\;(h)=\frac{0.693}{ke\left(1/h\right)}\\ \text{T}1/2\;(\text{h})=12.15. \end{array}$$

The differences in the primary and secondary pain scores between two groups were compared. Also, the mean number of patients’ consumed Acetaminophen tablets during ten days of treatment period was estimated and compared for the two groups. Patients in Group B took an average number of 5.08 rescue medication which was a bit greater compared to Group A (3.08) though it was not significant (p = 0.597). Also, no significant difference was observed between the difference of pain scores of groups after treatment and the number of Acetaminophen taken by Group A and Group B. Finally, the correlation between age, gender, and weight with pain score difference was investigated indicating no significant correlation.

## 4. Discussion

Sodium valproate is an anticonvulsant drug [10–28] widely used in treating various neuropathic pains induced by cancer, diabetes, or irritation of trigeminal nerve and also treating various headaches such as migraneous headaches [6–8, 29–32]. None of the existing studies have investigated the efficiency of valproate sodium in relieving radicular pains. Since the pathophysiological nature of these pains is different from that of other mentioned neuropathies, the results and findings of these studies could not be safely generalized to this type of pain and to this group of patients with this problem [5]. An important point to be kept in mind by physicians is the administration of the minimum effective dose which both satisfies patients and induces the least amount of complications in them. The incidence of a common but unpleasant complication such as gastrointestinal disturbances may result in patient dissatisfaction and cessation of their compliance with the physicians and required follow-up. The prescribed daily dose of VPA in all clinical trials conducted so far has been in the range of 1800–2400 mg/day [8]. The consumption of this amount of VPA per day would undoubtedly lead to many gastrointestinal and hepatic complications.

In this study, the group receiving VPA and Celecoxib simultaneously reported a maximum reduction of 79 units and a minimum reduction of 35 units in pain score (Table 3). Also, the group receiving placebo and Celecoxib simultaneously reported a maximum reduction of 74 units and a minimum reduction of 28 units in pain score. The P-values obtained indicate a significant difference between the primary and secondary pain scores in Groups A and B meaning that there has been a significant improvement. A cross-comparison of pain score reduction among the two groups suggested no significant difference. However, the fact that the pain showed an acceptable response to analgesics and anti-inflammatory drugs during initial days of treatment can’t be used as a basis for our judgment on the long run. Consequently, increasing the treatment length and longer patient follow-ups in future research can result in better and more valid results. Moreover, lack of a separate control group to receive placebo alone due to ethical codes of research prevented us from eliminating the psychological effect of drug consumption on our results. This finding shows separately the comparison of the pain scores before and after intervention in the two groups. As the plasma concentration of the drug alone does not indicate its efficiency in controlling convulsions [33], it is neither an appropriate index for judgments on controlling pain in patients. Nevertheless, this rate of pain reduction obtained with low doses of valproate sodium may be rendered as an improvement in pain reduction for patients who had occasionally reported severe pains.

In the study by Drews et al. (1994), 1200–2400 mg/ day valproate sodium was administered to 20 spinal injury patients on the basis of their response to treatment (the mean plasma concentration of the patients was 614 μmol/L). The response to treatment and pain reduction in patients were investigated by McGill pain questionnaire. No statistically significant difference was found at the end of the treatment period between the patients who received this medicine and those who received placebo despite a %33 reduction in pain [8].

Kochar et al., assessed the role of sodium valproate for treating of pain in diabetic neuropathy. In this regard, they assessed pain via Visual Analogue score McGill Questionnaire at beginning, after 1 and 3 months of study and observed that sodium valproate provides significant improvement in reducing pain in patients with diabetic neuropathy, which was consistent with our study [34]. Hardy et al., also observed the efficacy of sodium valproate in neuropathic pain of cancer patients [35]. Hering et al., assessed the role of sodium valproate in the treatment of headache. In this regard, they administered the dose 600 and 2000 mg/day for patients and reported that sodium valproate seems to be an efficacious drug for treatment of headache [36]. This finding was also consistent with our study. Cuter et al., estimated the effect of valproate in pain of animal models and reported that the effect of valproate is performed via binding to GABA receptor in animal models [37]. But, Gill et al., evaluated the role of Valproic acid for neuropathic pain during 12 weeks and reported that no adequate evidence was seen to use the Valproic acid [38]. It seems that more research should be done in this regard, especially Valproic acid and radicular pain.

In addition, clinical observations on our population race demonstrated that for some medicines, the doses administered to patients on the basis of medical references exceeded the needs of the patients. The clinical study has been done on Iranian race indicated that Iranian patients have different pharmacokinetic parameters of Phenytoin from the reported mean values in other population, according to this study; Iranian population demonstrated higher Michaelis-Menten constant (Km) and lower maximum rate of metabolism (Vm) than other races and ignoring this issue could lead to increasing risk of adverse reactions. E. Salehifar and et al claimed that our population may have a lower metabolic capacity for phenytoin metabolism and lower expression of CYP2C9 and CYP2C19 that these data could help the specialists to optimization and individualize antiepileptic therapy [39]. CYP-mediated biotransformation is one of the route of VPA metabolism and CYP2C9 play the pivotal role in that [40]. Thus according to the research has been done on 179 Asian patients for investigating influence of cytochrome oxidase polymorphisms on the steady-state standardized plasma VPA concentrations; subjects with low expression of CYP2C9 alleles had higher mean plasma valproic acid concentrations in comparison to those without, because these categories of metabolizing enzymes lead VPA to inactive metabolites such as 4-OH-VPA and 5-OH-VPA [41]. Another study conducted in Iran on the effects of Daclizumab in kidney transplant patients approved the efficacy of low dose (two doses) of the drug without noticeable complications while Daclizumab is routinely used with higher doses (five doses) to prevent transplant rejection [42]. This study provides us with an evidence of pharmacodynamic differences of drugs in the Iranian population. Since speed of metabolism and subsequently, inactivation of drugs plays a significant role in determining the effective required dose, hence, a revision of the current doses of some drugs is rendered as an important issue in drug therapy in various races of patients.

Also, in surveying the number of Acetaminophen tablets taken by each group, it was observed that, patients in Group A had taken 3.08 ± 5.16 tablets on average while those in Group B had consumed 5.08 ± 8.07 Acetaminophen tablets on average being greater than Group A. The two groups under study were not significantly different in this parameter (p value = 0.597). It should be pointed out that during the orientation session when the procedures of drug administration were explained to each patients, it was observed that the majority of patients had a negative attitude towards Acetaminophen consumption showing no inclination for taking this tablet even in the presence of pain. This can justify the low mean of Acetaminophen consumption in the Placebo Group and even the intervention group. The pharmacokinetic parameters obtained from the intervention group receiving VPA are significantly different from those obtained for population studies (Table 4). These statistical data demonstrated that drug clearance of our patients is lower than that of in population studies, also the similar results have been reported in Sohrevardi SM et al [42].

This indicates that our study population demands its own pharmacodynamic and pharmacokinetic investigations and that making judgments on the basis of studies on other populations would be misleading and inaccurate. The half-life obtained from kinetic estimations for the consumption of the drug once per day was averagely 29.6 h for its simultaneous consumption with Celecoxib (Table 2), while the half-life reported for this drug in adults in pharmacopoeia is 5–20 h [18, 43].

A wide fluctuation was observed in trough concentration among the patients in Group A so that an SD of 13.53 was obtained for this group. This could be probably attributed to the effect of Celecoxib in the metabolism and clearance of drug which varies from individual to individual (Table 2). An increased drug clearance may also be used as the basis for judgments on drug elimination.

Chronic pains such as pains of neuropathic nature may have several simultaneous causes, all of which are able to play a role in the course of patient’s healing. Consequently, these pains may be considered to be multifactorial among which are anxiety disorders, depression, insomnia, and low energy levels or lethargies. Hence, chronic pain control is not possible just with eliminating the inducing factor, rather it demands some multilateral approach [44]. Moreover, the effects of environmental factors, nutritional habits, life style, age, and health level on individuals’ responses to drugs could not be ignored since they alter the results of kinetic studies. On the other hand, the genetics of individuals also affects the kinetics and dynamics of drugs as metabolizing enzymes of drugs, pharmaceutical receptors, protein transmitters of drugs, ionic channels, etc are controlled by genes. Thus, the effects of genetics, race, and life environment are of utmost importance in the incidence of pharmacological complications and treatment responses [45].

## 5. Conclusion

On the basis of the interventions conducted in this study, we may conclude that:

1. Low doses of valproate sodium specifically along with NSAIDs demonstrate very pronounced therapeutic effects on reduction and even removal of radicular chronic pains.
2. The half-life, elimination constant and true clearance of drug for each patient was significantly different from the equivalent parameters obtained from population studies (Table 4).
3. Lower true clearance in our patients in comparison of population studies results proved that not only drug efficacy assessment but also therapeutic drug monitoring (TDM) and drug plasma concentration determination is necessary for prescription.

Hence, it is recommended that supplementary studies on pharmacokinetics and their NSAIDs interactions be carried out with a focus on eliminated metabolites of drugs.

## Figures and Tables

**Table 1:** Distribution of males and females in terms of parameters.

Group	parameters						

	Age	Weight (Kg)	VAS 1 (mm)	VAS 2 (mm)	Pain area		

					C^a^	L^b^	L-C
A	44.8 ± 14.9	71.2 ± 12.1	58.5 ± 24.4	36.6 ± 25.8	12 (31.6%)	24 (63.2%)	2 (5.3%)
B	48.5 ± 12.9	71.6 ± 11.4	49.0 ± 21.2	34.6 ± 23.5	8 (21.1%)	29 (76.3%)	1 (2.6%)
P value	**NS**^c^	**NS**	**NS**	**NS**	NS		

a Cervical.

b Lumbar.

c Not significant.

**Table 2:** Pharmacokinetic parameters in group A.

Group	Conc. (mg/L)	K_e_(1/h)	AUC	V_d_ (L/kg)	Cl_true_ (ml/kg/h)	Cl_population_ (ml/kg/h)	t_1/2_ (h)
A	26.9 ± 13.53	0.02 ± 0.01	15.54 ± 7.95	9.73 ± 1.59	0.26 ± 0.09	0.55 ± 0.09	29.65 ± 14.31

**Table 3:** Comparison of various pain scores in groups.

Group	VAS 1 (mm)	VAS 2 (mm)	Mean (mm) VAS2-VAS1	P value
A	58.5 ± 24.4	36.6 ± 25.8	−21.9±25.4	<0.001
B	49.0 ± 21.2	34.6 ± 23.5	−14.3±23.0	<0.001

**Table 4:** Comparison of true and population pharmacokinetic parameters in group A.

			P value
**Group A**	**CL_true_**	0.26 ± 0.09	<0.001
	**CL_population_**	0.55 ± 0.09	
	**T_1/2true_**	29.65 ± 14.31	<0.001
	**T_1/2 population_**	12.15	
	**K_etrue_**	0.027 ± 0.01	<0.001
	**K_e population_**	0.057	
